# Organelle Crosstalk in Renal Cells: Insights from Cell Biology and Implications for AKI-to-CKD Transition

**DOI:** 10.3390/ijms27125207

**Published:** 2026-06-09

**Authors:** Rossana Franzin, Monica Campioni, Anna Storelli, Gabriele Ruggieri, Sabrina Molino, Giorgio Ladisa, Anna Gallone, Marco Fiorentino, Loreto Gesualdo, Paola Pontrelli

**Affiliations:** 1 Nephrology, Dialysis, and Transplantation Unit, Department of Precision and Regenerative Medicine and Ionian Area, University of Bari Aldo Moro, 70124 Bari, Italy; 2 Department of Translational Biomedicine and Neuroscience (DiBraiN), University of Bari Aldo Moro, 70121 Bari, Italy

**Keywords:** organelle crosstalk, cGAS-STING axis, unfolded protein response (UPR), autophagic flux, proteostasis, mitochondrial dysfunction, PEX genes, membrane contact sites (MCSs), ER-mitochondria-associated membranes (MAMs), MFN2-PERK axis

## Abstract

The kidney is a highly specialized organ that maintains systemic homeostasis through tightly coordinated cellular and molecular mechanisms. Renal parenchymal cells regulate metabolic waste excretion, electrolyte and acid–base balance, and blood pressure control—functions that rely on the dynamic integration of intracellular organelles. Recent advances in molecular and biochemical research have highlighted how inter-organelle communication is essential for preserving renal cell function and adaptive responses to stress. This review focuses on the molecular crosstalk among key organelles—including the nucleus, endoplasmic reticulum (ER), Golgi apparatus, mitochondria, lysosomes, and peroxisomes—primarily in tubular epithelial cells. We discuss how these interactions coordinate metabolic signaling, protein homeostasis, redox balance, and energy production and how their disruption contributes to maladaptive pathways during acute kidney injury (AKI), ultimately promoting chronic kidney disease (CKD) transition. Particular focus is placed on emerging pathways linking organelle dysfunction to inflammation, fibrosis, and metabolic reprogramming. Furthermore, we highlight recent advances in genetics and molecular therapeutics targeting organelle communication, including modulation of ER stress responses, mitochondrial biogenesis, and lysosomal function. Clinically approved agents, such as mTOR inhibitors, and experimental approaches—such as chemical chaperones and mitochondrial transplantation—demonstrate the potential to restore organelle homeostasis and mitigate renal injury. Overall, elucidating the molecular networks governing organelle crosstalk provides critical insights into kidney disease pathogenesis and identifies novel targets for therapeutic intervention in AKI-to-CKD transition.

## 1. Introduction

In the eukaryotic cells, membrane-bound organelles form a layer of compartmentalization essential to isolate biochemical environments, accelerate reactions by increasing metabolic intermediates, or separate dangerous substances such as proteolytic enzymes. Traditionally, organelles were viewed as discrete entities communicating indirectly via cytosolic diffusion or vesicular trafficking [1]. This paradigm has been revised with the recognition that organelles form direct physical interactions through membrane contact sites (MCSs), where opposing membranes are closely apposed without fusion. These contacts are maintained by protein complexes that tether adjacent membranes, enabling rapid, non-vesicular exchange of lipids, ions, and metabolites and thereby coordinating cellular homeostasis [2]. Collectively, these interaction networks define an additional organizational layer, the “contactome”, complementing other omics-scale levels such as the transcriptome, proteome, and metabolome [3]. Technological advances, including transmission electron microscopy and fluorescence-based organelle labeling, have further refined our understanding of intracellular organization [4]. Organelles are now considered spatially heterogeneous structures composed of functionally specialized subdomains, or “organelle zones,” broadly categorized into response, communication, and sorting domains (i.e., areas responsible for cargo sorting and trafficking) [3]. Among these, MCSs represent key communication hubs that integrate signals and metabolic flux across organelles.

Biochemical evidence of such interactions emerged with the identification of mitochondria-associated membranes (MAMs), specialized endoplasmic reticulum (ER) subdomains closely opposed to the outer mitochondrial membrane (OMM) at a distance of approximately 10–30 nm [5]. MAMs are dynamic platforms regulated by multiprotein complexes that control their structure and function, enabling coordination of calcium signaling, lipid metabolism, stress responses, mitochondrial dynamics, and autophagy with a crucial involvement in neurogenerative diseases [6].

This level of integration is also critical in renal tubular epithelial cells, which operate in a highly energy-demanding and metabolically complex environment [7]. Maintenance of epithelial polarity, transport functions, and metabolic flexibility relies on coordinated activity among the nucleus, ER, mitochondria, Golgi apparatus, lysosomes, and peroxisomes. These organelles communicate through MCSs and shared signaling pathways that regulate calcium homeostasis, lipid trafficking, proteostasis, and redox balance, such that nephron function emerges from their dynamic interplay rather than from isolated organelle activity.

Recent studies have highlighted how this intracellular network supports renal cell recovery. ER–mitochondria interactions regulate calcium transfer and bioenergetics; lysosomes integrate degradative and nutrient-sensing pathways through autophagy; and peroxisomes cooperate with both ER and mitochondria in oxidative metabolism and lipid remodeling. Disruption of this coordination—through mitochondrial dysfunction [8], ER stress, or impaired autophagy—propagates across organelle networks, amplifying oxidative stress, inflammation, and defective epithelial repair.

Within this framework, kidney injury can be viewed as a failure of organelle integration. This is particularly relevant in acute kidney injury (AKI) [9] and chronic kidney disease (CKD) [10], now recognized as interconnected conditions rather than distinct entities.

AKI is defined as a rapid decline in kidney function occurring over hours up to seven days, typically identified by an increase in serum creatinine and/or reduced urine output [11], whereas CKD is characterized by persistent abnormalities in kidney structure or function lasting for more than three months [12]. AKI commonly arises from ischemia/reperfusion injury (IRI), sepsis, nephrotoxic drugs, or major surgery [9], while CKD is most frequently associated with diabetes mellitus, hypertension, glomerular diseases, and aging. Epidemiologically, AKI affects approximately 10–15% of hospitalized patients and over 50% of critically ill individuals, whereas CKD affects nearly 10% of the global population and represents a growing cause of morbidity and mortality worldwide [9,11,12]. Increasing evidence indicates that AKI and CKD are mechanistically linked, with episodes of AKI accelerating CKD progression and pre-existing CKD predisposing patients to recurrent AKI events [13]. In this context, the transition from AKI to CKD can be interpreted as a progressive failure of organelle homeostasis and cellular adaptation [14,15] leading to maladaptive repair [16]. Proposed mechanisms include nephron loss, endothelial dysfunction with vascular rarefaction and hypoxia, epigenetic alterations, tubular cell-cycle arrest, and persistent inflammation [17]. These processes converge on a chronic stress state often characterized by sustained hypoxic signaling and ER stress, indicating that prolonged organelle dysfunction is a central determinant of disease progression.

Early alterations in mitochondrial metabolism, ER proteostasis, and lysosome-dependent quality control impair the adaptive capacity of tubular epithelial cells, shifting the balance from regeneration toward fibrosis and dysfunction. Elucidating these mechanisms at the level of organelle crosstalk is therefore essential to bridge fundamental cell biology with kidney pathophysiology.

Here, we synthesize current knowledge on the molecular and structural basis of organelle interplay in renal cells, with a focus on nucleus–mitochondria communication, ER–MAMs, lysosome–autophagy pathways, peroxisomal metabolism, and organelle contact sites as regulatory hubs. Understanding how these interconnected systems govern cellular fate may provide new opportunities to limit AKI and its progression to CKD.

## 2. Nucleus

**Figure 1:**
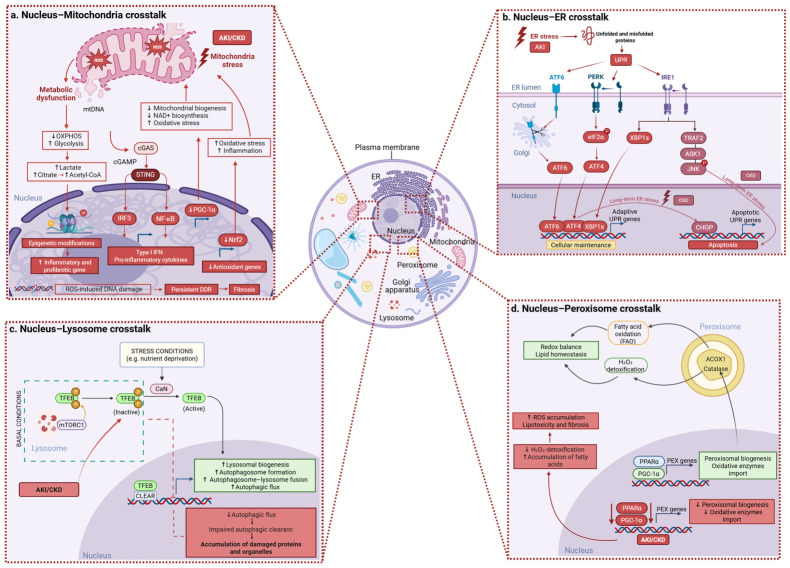
(**a**) **Nucleus–Mitochondria crosstalk**. Mitochondria–nucleus communication coordinates metabolic and inflammatory signaling: mitochondrial damage in AKI promotes mtDNA release and activation of the cGAS–STING pathway, promoting IRF3- and NF-κB-dependent inflammation, while PGC-1α downregulation impair mitochondrial function and Nrf2 attenuation exacerbates oxidative stress and fibrosis in CKD. Mitochondrial metabolic dysfunction further promotes epigenetic remodeling through glycolytic reprogramming and accumulation of lactate and citrate/acetyl-CoA, contributing to inflammatory and profibrotic gene expression. In parallel, oxidative stress induces activation of DNA damage response (DDR) pathways, which may support DNA repair and cell survival or, when persistently activated, promote maladaptive repair and fibrosis progression. (**b**) **Nucleus–ER crosstalk**. The ER–nucleus axis is mediated by UPR, whereby ATF6, PERK, and IRE1 transmit proteostatic signals to the nucleus to restore cellular homeostasis in AKI; under persistent ER stress, in CKD, this adaptive crosstalk becomes maladaptive, driving CHOP-dependent apoptosis and contributing to fibrosis and disease progression. (**c**) **Nucleus–Lysosome crosstalk**. The lysosome–nucleus axis, governed by TFEB, coordinates lysosomal biogenesis and autophagic clearance in response to cellular stress; in AKI/CKD, impaired TFEB activation reduces autophagic flux and lysosomal clearance, leading to accumulation of damaged proteins and organelles. (**d**) **Nucleus–Peroxisome crosstalk**. Bidirectional signaling between nucleus and peroxisomes, coordinated by the PPARα/PGC-1α axis, sustains peroxisomal biogenesis, fatty acid oxidation, and redox balance; disruption of this transcriptional program during renal injury impairs PEX gene expression and antioxidant capacity, promoting ROS accumulation, lipotoxicity, and fibrosis. Blue and black lines: physiological conditions; red lines: AKI, AKI-to-CKD conditions. Abbreviations: AKI: acute kidney injury; CKD: chronic kidney disease; ROS: reactive oxygen species; mtDNA: mitochondrial DNA; OXPHOS: oxidative phosphorylation; NAD^+^: nicotinamide adenine dinucleotide; cGAS: cyclic GMP-AMP synthase; cGAMP: cyclic GMP-AMP; STING: stimulator of interferon genes; IRF3: interferon regulatory factor 3; NF-κB: nuclear factor kappa B; PGC-1α: peroxisome proliferator-activated receptor gamma coactivator 1-alpha; Nrf2: nuclear factor erythroid 2-related factor 2; Type I IFN: type I interferons; DDR: DNA damage response; UPR: unfolded protein response; ATF6: activating transcription factor 6; PERK: protein kinase RNA-like endoplasmic reticulum kinase; IRE1: inositol-requiring enzyme 1; eIF2α: eukaryotic initiation factor 2 alpha; ATF4: activating transcription factor 4; XBP1s: X-box binding protein 1; TRAF2: TNF receptor-associated factor 2; ASK1: apoptosis signal-regulating kinase 1; JNK: c-Jun N-terminal kinase; CHOP: C/EBP homologous protein; TFEB: transcription factor EB; mTORC1: mechanistic target of rapamycin complex 1; CaN: calcineurin; CLEAR: coordinated lysosomal expression and regulation; FAO: fatty acid oxidation; ACOX1: Acyl-CoA oxidase 1; PPARα: peroxisome proliferator-activated receptor alpha. Figures were created with BioRender.com.

The nucleus is a membrane-bound organelle that encloses the genomic DNA organized as chromatin and constitutes the principal site of DNA replication and transcriptional control in eukaryotic cells. Proximal tubular epithelial cells (PTECs) are characterized by eccentric, round-to-oval nuclei, with highly metabolically active cells often containing prominent nucleoli [18]. Distinct, heterogenous gene expression profiles exist in nuclei across S1 (convoluted), S2, and S3 (straight/medullary) segments in health and renal disease [19]. S1 cells in the renal cortex primarily mediate bulk reabsorption of glucose, amino acids, bicarbonate, and low-molecular weight proteins and therefore show enrichment of genes involved in high-capacity, energy-dependent transport. S2 cells retain reabsorptive functions but are more enriched in metabolic and detoxification pathways, including xenobiotic handling and organic ion transport. In contrast, S3 cells in the outer medulla operate in a relatively hypoxic environment and preferentially express genes related to anaerobic metabolism and stress responses, which also contributes to their higher susceptibility to ischemic and toxic injury in AKI [19]. Podocytes, by contrast, are highly specialized, terminally differentiated epithelial cells of the glomerulus, distinguished by large, often elongated nuclei that extend toward Bowman’s space [20]. In PTECs, nuclear transcriptional programs sustain mitochondrial biogenesis and fatty acid oxidation (FAO), the dominant metabolic pathway supporting the high energetic demands of active solute transport primarily through peroxisome proliferator-activated receptor gamma coactivator 1-alpha (PGC-1α)-dependent regulation [21,22] (Figure 1a). By coordinating the expression of genes required for mitochondrial biogenesis and metabolic function, the nucleus supports the elevated metabolic requirements of the renal cortex [23]. In glomerular podocytes, maintenance of the differentiated state required for filtration barrier integrity depends on tightly regulated transcriptional networks, including super-enhancers (large, high-density clusters of regulatory DNA elements that drive robust gene expression), which control key podocyte identity factors such as FOXC1 and FOXC2 [24]. Accordingly, FOXC1/2 cooperate to maintain podocyte differentiation by regulating the expression of genes such as podocin and C-X-C motif chemokine ligand 12 (Cxcl12) [25]. Through the regulation of adaptive gene expression programs controlling inflammation, metabolism, and cell survival, nuclear signaling pathways influence the capacity of renal cells to recover from injury [26].

### 2.1. Role of Nucleus in AKI-to-CKD

Renal injury is characterized by coordinated activation and reprogramming of nuclear signaling pathways that sense genotoxic stress and regulate transcriptional responses. A central early event is the induction of oxidative stress, which promotes activation of the nuclear DNA damage response (DDR). In renal cells, in DNA lesions induced by reactive oxygen species (ROS), nephrotoxins such as cisplatin or IRI activate key DDR kinases, including ATM (ataxia telangiectasia mutated) and ATR (ATM- and Rad-3-related) [27]. ATM is primarily activated by DNA double-strand breaks, where it is recruited and phosphorylates histone H2AX, promoting chromatin remodeling and the recruitment of DNA repair proteins, as well as activation of downstream signaling through checkpoint kinase 2 (Chk2) and p53, ultimately regulating DNA repair, cell cycle arrest, and cell fate decisions [28].

In AKI, the DDR exerts a dual and context-dependent role by integrating adaptive and maladaptive responses. The specific outcome is determined by the type and intensity of injury and by the dominant upstream damage signals. In murine and tubular epithelial cell models of cisplatin-induced nephrotoxicity, DDR signaling is mainly pro-apoptotic, with strong activation of ATR-dependent pathways that converge on Chk2 and p53, ultimately driving tubular epithelial cell apoptosis [29]. In contrast, studies in murine models of IRI and in vitro models of ATP depletion/recovery in proximal tubular cells suggest that ATM activation appears to play a predominantly cytoprotective role by promoting DNA repair, although p53 activation still contributes to tubular cell apoptosis. More broadly, depending on AKI severity and stage, DDR can induce either pro-survival programs (DNA repair, transient cell cycle arrest, and autophagy) or pro-death pathways. However, when DNA damage persists and DDR signaling remains chronically activated, tubular cells undergo sustained G2/M cell cycle arrest, leading to impaired regenerative capacity and the acquisition of a pro-fibrotic secretory phenotype that promotes maladaptive repair and progression toward CKD [30]. However, the extent to which this profibrotic program is conserved in human AKI-to-CKD transition remains incompletely defined [31].

In addition to DDR pathways, DNA damage also activates poly(ADP-ribose) polymerase 1 (PARP1), an NAD^+^-dependent enzyme involved in DNA repair processes. While PARP1 activity contributes to genomic maintenance under physiological conditions, its hyperactivation during severe stress leads to excessive NAD^+^ consumption, amplifying metabolic stress and nuclear signaling dysfunction. Experimental studies using primary cultures of rat proximal tubular cells exposed to oxidative stress demonstrated that PARP1 activation contributes to mitochondrial dysfunction and tubular cell injury following ROS-induced DNA damage [32]. These findings further support the concept that nuclear stress responses contribute to metabolic dysfunction during renal injury. Again, most of the available evidence comes from experimental models, and direct validation in human kidney tissue is still limited [33].

Beyond DDR pathways, nuclear responses to renal injury are also critically shaped by redox-sensitive transcriptional programs. Oxidative stress activates nuclear factor erythroid 2-related factor 2 (Nrf2) (Figure 1a), a redox-responsive transcription factor that translocates to the nucleus and induces the expression of antioxidant and cytoprotective genes, including NAD(P)H: quinone oxidoreductase 1 (NQO1), heme oxygenase-1 (HO-1), and enzymes involved in glutathione metabolism [34,35]. Within this framework, Nrf2 represents a key adaptive transcriptional axis that counterbalances oxidative and inflammatory signaling. However, when injury persists, nuclear signaling networks progressively shift toward stable activation of inflammatory and profibrotic transcriptional programs. In CKD, the protective Nrf2 pathway becomes progressively attenuated. Although Nrf2 signaling initially promotes cytoprotective responses during AKI, advanced CKD is frequently associated with impaired nuclear activation of Nrf2, leading to reduced antioxidant transcriptional capacity and amplification of oxidative and inflammatory stress [36]. At the same time, profibrotic signaling pathways become persistently activated in the nucleus of injured tubular epithelial cells. In particular, transforming growth factor-β (TGF-β) signaling promotes phosphorylation and nuclear accumulation of Smad2/3 complexes, which act as transcriptional regulators of genes involved in extracellular matrix production, myofibroblast activation, and fibrogenic reprogramming [37,38]. Sustained nuclear Smad3 activity stabilizes profibrotic transcriptional networks and contributes to progressive renal dysfunction, highlighting the TGF-β/Smad pathway as a central determinant of maladaptive repair [37,39].

### 2.2. Crosstalk with Nucleus

In kidney diseases, nucleus–organelle crosstalk represents a central pathogenic mechanism through which stress signals from mitochondria, the ER, lysosomes, and peroxisomes converge to modulate inflammatory and metabolic transcriptional programs.

*Nucleus–Mitochondria Crosstalk*

Multiple pathogenic stimuli, including IRI, metabolic alterations, oxidative stress, and cellular senescence, induce mitochondrial damage and the subsequent release of mitochondrial DNA (mtDNA) into the cytosol. Acting as a damage-associated molecular pattern (DAMP), mtDNA activates the innate immune cyclic guanosine monophosphate-adenosine monophosphate (GMP-AMP) synthetase (cGAS)–stimulator of interferon gene (STING) signaling pathway [40]. While this mechanism is essential for host defense, its aberrant activation promotes inflammatory responses that contribute to the onset and progression of renal diseases, including AKI and CKD [41]. In AKI, mitochondrial damage leads to increased membrane permeability and the cytosolic leakage of mtDNA [40,41]. Once in the cytosol, mtDNA is sensed by cGAS, which catalyzes the production of the second messenger 2′3′-cyclic GMP-AMP (cGAMP), which in turn activates the adaptor protein STING and promotes downstream signaling through TANK-binding kinase 1 (TBK1), leading to activation of interferon regulatory factor 3 (IRF3) and the expression of proinflammatory factors such as type I interferons (IFN-I) [42]. The oligomerization of STING also promotes the activation of the IκB kinase (IKK), which triggers NF-κB signaling and drives the expression of proinflammatory mediators such as TNF-α, IL-6, and MCP-1, ultimately amplifying tubular inflammation and injury [41,43,44]. Beyond its role in acute inflammation, sustained activation of the cGAS–STING axis contributes to maladaptive repair and disease progression (Figure 1a). Persistent signaling promotes immune cell recruitment, fibroblast activation, and extracellular matrix deposition, driving the transition from AKI to CKD and renal fibrosis [43].

Experimental evidence further supports a pathogenic role for mtDNA-mediated inflammatory signaling in AKI. In a murine model of cisplatin-induced AKI, Maekawa et al. demonstrated that cisplatin-induced mtDNA leakage into the cytosol of tubular epithelial cells activated the cGAS–STING pathway, thereby promoting renal inflammation and disease progression [40,41]. Consistent with these experimental findings, elevated urinary mtDNA levels have also been detected in patients with sepsis-associated AKI and correlate with markers of renal dysfunction and tubular injury, including plasma creatinine, neutrophil gelatinase-associated lipocalin (NGAL), and kidney injury molecule-1 (KIM-1) [45]. Persistent activation of the cGAS–STING pathway has been associated with renal inflammation and fibrotic remodeling following kidney injury (Figure 1a). Accordingly, studies in murine models demonstrated that genetic or pharmacological inhibition of STING attenuates kidney injury and fibrosis, supporting a pathogenic role for sustained cGAS–STING signaling in maladaptive renal repair [46].

Beyond ROS-mediated nuclear stress responses, mitochondrial metabolic dysfunction may also drive epigenetic reprogramming in kidney disease. In AKI and during AKI-to-CKD transition, impaired oxidative phosphorylation promotes a glycolytic shift associated with lactate accumulation in renal tubular epithelial cells [47]. Lactate has recently emerged as a signaling metabolite capable of regulating gene expression through histone and non-histone lactylation, thereby contributing to inflammatory and profibrotic responses in kidney disease [48,49]. In experimental models of septic AKI, H3K18 lactylation was associated with activation of NF-κB signaling, promoting inflammation, apoptosis, and renal dysfunction [50]. Similarly, elevated H4K12 lactylation levels in CKD patients and murine models positively correlated with renal inflammation and fibrosis [51]. In addition, non-histone lactylation has been linked to mitochondrial fragmentation and maladaptive repair responses in septic AKI [52]. Beyond lactate-mediated lactylation, disrupted mitochondrial metabolism may also promote epigenetic changes through altered citrate and acetyl-CoA metabolism. In murine models of CKD-associated renal fibrosis, citrate accumulation and increased ATP-citrate lyase (ACLY)-dependent acetyl-CoA production were associated with enhanced H3K27 acetylation and activation of inflammatory and profibrotic transcriptional programs. Importantly, these alterations were also observed in human CKD kidneys and correlated with worsening renal function [53]. Overall, the mechanistic relevance of these epigenetic pathways is strongly supported by preclinical data, whereas their relative contribution in human AKI-to-CKD remains less well established.

Mitochondrial dysfunction also impairs transcriptional programs involved in mitochondrial biogenesis and metabolic homeostasis of tubular cells. In kidney diseases, PGC-1α expression and activity are consistently downregulated across different pathological contexts, including AKI and CKD. This reduction is driven, at least in part, by inflammatory and profibrotic signaling pathways, such as NF-κB, TNF-α, TWEAK, and TGF-β, thereby establishing a direct link between inflammatory signaling and impaired mitochondrial homeostasis [22,54]. In AKI, experimental models of cisplatin nephrotoxicity, sepsis-associated AKI, and IRI demonstrated marked downregulation of PGC-1α expression, associated with impaired mitochondrial biogenesis and dysfunction, leading to increased oxidative stress, tubular cell apoptosis, and increased susceptibility to injury. In CKD, persistent repression of the PGC-1α axis contributes to sustained mitochondrial dysfunction, impaired FAO, and lipid accumulation, as demonstrated in both human CKD and experimental models of renal fibrosis.

However, the association between reduced therapeutic reversibility and the quantitative relevance of this pathway in human disease remains incompletely established.

*Nucleus–ER crosstalk*

Mitochondrial signaling is crucial; furthermore, ER stress represents another key pathway of nucleus–organelle communication in renal disease. The structural continuity between the outer nuclear membrane and the ER provides a platform for the direct communication between these compartments. A key mediator of this crosstalk is the unfolded protein response (UPR), which is activated in response to the accumulation of misfolded proteins within the ER lumen. Under these conditions, the ER chaperone GRP78 dissociates from the transmembrane sensors activating transcription factor 6 (ATF6), inositol-requiring enzyme 1α (IRE1α), and protein kinase R-like ER kinase (PERK), leading to their activation. UPR activation enhances the protein-folding capacity of the ER by increasing the expression of ER-resident chaperones, reducing protein synthesis, and promoting degradation of misfolded proteins through ER-associated degradation (ERAD) [55] (Figure 1b).

In renal cells, UPR activation has been implicated in both AKI and CKD [56]. In AKI, ER stress induced by accumulation of unfolded and misfolded proteins leads to activation of UPR pathways aimed at restoring protein homeostasis. In chronic conditions, sustained ER stress and prolonged UPR activation are associated with increased inflammation and fibrosis. UPR signaling also contributes to activation of inflammatory pathways, including NF-κB, and promotes production of pro-inflammatory cytokines. Moreover, persistent ER stress is linked to epithelial-to-mesenchymal transition and extracellular matrix deposition, contributing to tubulointerstitial fibrosis and progressive renal dysfunction [56].

However, most available evidence derives from preclinical models, and the extent to which these ER stress pathways are quantitatively conserved in human AKI-to-CKD remains incompletely defined [57].

*Nucleus–Lysosome*

The nucleus and the lysosome engage in a sophisticated feedback loop that regulates cellular clearance. The central mediator of this axis is the transcription factor EB (TFEB). Under basal conditions, TFEB is sequestered in the cytoplasm by mammalian target of rapamycin complex 1 (mTORC1)-mediated phosphorylation on the lysosomal surface. When the cell encounters nutrient deprivation or lysosomal stress—common in various nephropathies—TFEB is dephosphorylated and rapidly translocates to the nucleus. Its activity is controlled by phosphorylation by kinases such as mTOR and ERK, which retain it in the cytoplasm in an inactive form.

Experimental studies in septic AKI indicate that TFEB-dependent lysosomal responses are impaired, leading to defective autophagic degradation and tubular injury. These data support the idea that altered kinase signaling may affect TFEB localization and activity in AKI, although the precise contribution of mTOR- and ERK-mediated TFEB cytoplasmic retention has not been directly established in a single kidney-specific model [58,59,60] (Figure 1c).

*Nucleus–Peroxisome*

The metabolic efficiency of renal tubular cells depends on continuous bidirectional communication between the nucleus and peroxisomes. In the healthy kidney, the nucleus acts as the master regulator of peroxisomal fitness via the PPARα/PGC-1α axis [21]. This signaling pathway orchestrates the de novo biogenesis of the organelle by inducing the transcription of PEX genes (peroxins), which are essential for membrane assembly and the import of key oxidative enzymes like Acyl-CoA oxidase 1 (ACOX1) and catalase. This coordinated program ensures effective fatty acid processing and redox balance, preventing lipotoxic damage in the proximal tubule [61] (Figure 1d).

During renal injury, this vital axis undergoes transcriptional repression documented in several experimental models of AKI. In AKI, this process has been described in cisplatin-induced AKI, where mouse kidneys and LLC-PK1 tubular cells showed reduced PGC-1 expression, impaired PPARα DNA-binding activity, and suppression of fatty acid oxidation and peroxisomal metabolism [62]. In the same model, PPARα agonist treatment restored fatty acid oxidation and attenuated acute renal failure [63]. Similar downregulation of the PGC-1α axis was also observed in sepsis-associated AKI and ischemia-reperfusion models, where it was linked to worse mitochondrial dysfunction and renal injury. The inability of the nucleus to re-establish the PEX-mediated peroxisomal machinery causes the accumulation of non-oxidized fatty acids, fueling pro-fibrotic gene expression [64]. Although these data support a role for nuclear control of peroxisomal metabolism in AKI, the contribution of PEX genes to human AKI-to-CKD progression remains incompletely defined [21,65].

### 2.3. Therapeutic Potential Approach

Preserving nuclear integrity and precisely modulating DDR signaling have emerged as key therapeutic strategies to limit AKI and restrict its progression toward chronic disease states. Experimental studies in animal models show that pharmacological inhibition of p53 or PARP1 markedly reduces tubular cell death and subsequent fibrotic remodeling compared with controls [4,8]. In parallel, increasing the availability of nicotinamide (NAM), a critical precursor of NAD^+^, enhances nuclear repair capacity and dampens activation of the pro-apoptotic p53 signaling axis [66].

Targeting redox-responsive nuclear transcriptional programs represents an additional mechanistic approach. Experimental evidence indicates that strategies designed to 9enhance Nrf2/antioxidant response element (ARE) signaling confer a nephroprotective effect by attenuating oxidative stress and inflammatory injury. Upon activation, Nrf2 translocates to the nucleus and coordinates an antioxidant transcriptional program that neutralizes ROS while functionally counteracting NF-κB signaling, thereby limiting the production of pro-inflammatory cytokines at their source [19,36,67,68,69]. In sepsis-associated AKI, NRF2 activation has been shown to preserve mitochondrial homeostasis and improve mitochondrial function, while in intravascular hemolysis-induced AKI, it reduces oxidative stress, inflammation, and tubular injury. In parallel, activation of the PGC-1α/TFEB axis has been shown to improve mitochondrial function and autophagic clearance in cisplatin-induced AKI, supporting the idea that restoring transcriptional control of organelle quality can favor renal recovery. More broadly, experimental and translational studies suggest that organelle crosstalk is a mechanistically attractive therapeutic target, and modulation of pathways linked to autophagy, mitochondrial biogenesis, and lysosomal function may help preserve cellular homeostasis during renal injury. However, most available therapeutic data derive from preclinical studies, and the clinical efficacy of these interventions in preventing human AKI-to-CKD progression remains to be established [23,66].

## 3. The Endoplasmic Reticulum (ER)

The endoplasmic reticulum (ER) is an extensive and dynamic network of interconnected membranous tubules and flattened sacs (called cisternae) that permeate the cytoplasm of eukaryotic cells. It accounts for more than 10% of total cell mass and nearly 60% of the total membrane system, making it one of the largest intracellular organelles. Morphologically and functionally, the ER is divided into rough ER (RER), characterized by ribosomes attached to its cytosolic surface, and smooth ER (SER), which lacks ribosomes [70].

Beyond its architectural complexity, the ER is organized into specialized subdomains that maintain distinct pH, redox conditions, and calcium concentrations. This compartmentalization enables the ER to finely regulate protein folding, lipid biosynthesis, and calcium storage and release. In renal cells, particularly tubular epithelial cells, the ER sustains the intense metabolic and transport demands required for electrolyte balance, acid–base regulation, nutrient reabsorption, and waste excretion [56,71]. In the kidney, the ER forms an extensive network in PTECs, where it sustains the high demand for the synthesis and trafficking of transporters and proteins involved in solute reabsorption and acid–base homeostasis [72]. At the cellular level, to preserve proteostasis, the ER activates a highly conserved signaling network known as the UPR when misfolded or unfolded proteins accumulate within its lumen. Under basal conditions, the three principal UPR sensors (PERK, IRE1α, and ATF6) are maintained in an inactive state through association with chaperone proteins such as BiP/GRP78. Accumulation of misfolded proteins triggers dissociation of binding immunoglobulin protein (BiP), allowing activation of these transmembrane sensors. However, in stress conditions, the UPR initially promotes adaptive signaling aimed at restoring ER homeostasis. Following different pathways, PERK attenuates global protein translation via eukaryotic translation initiation factor 2 subunit alpha (eIF2α) phosphorylation while selectively inducing ATF4; IRE1α mediates unconventional splicing of XBP1 mRNA, enhancing transcription of genes involved in chaperone production, ER-associated degradation (ERAD), and lipid biosynthesis. Finally, ATF6 translocates to the Golgi apparatus, where it undergoes proteolytic activation and subsequently induces expression of chaperones and ER quality control proteins.

However, when ER stress is severe or prolonged, the UPR transitions from a pro-survival to a pro-apoptotic program. Sustained PERK–ATF4 signaling induces CHOP, which promotes oxidative stress, mitochondrial dysfunction, and activation of apoptotic cascades. Prolonged IRE1α activation may also engage pro-inflammatory and apoptotic pathways through JNK signaling.

### 3.1. Role of Endoplasmic Reticulum in Renal Pathogenesis

The first evidence linking ER stress to AKI dates back to 1996, when ischemic renal injury was shown to induce the upregulation of ER chaperones involved in secretory protein folding. Subsequent studies demonstrated transient phosphorylation of PERK and eIF2α following IRI and cardiac arrest-associated AKI, establishing activation of the UPR as an early event in ischemic renal damage [73,74].

A wide range of renal stressors—including sepsis, oxidative stress, dysregulated iron and calcium homeostasis, viral infection, IRI, and exposure to nephrotoxic agents such as cisplatin or cadmium—promote the accumulation of misfolded proteins within the ER lumen, thereby triggering UPR signaling pathways. During the early phases of AKI, transient UPR activation exerts predominantly protective effects by attenuating global protein translation, enhancing ER folding capacity and promoting adaptive metabolic responses. In this context, ER stress signaling supports tubular epithelial cell survival.

In contrast, sustained or unresolved ER stress leads to prolonged activation of PERK–ATF4–CHOP and IRE1α pathways, which is associated with mitochondrial dysfunction, cytochrome c release, caspase activation, and apoptotic death of tubular epithelial cells. UPR signaling additionally modulates vascular responses through upregulation of the VEGFA–VEGFR2 axis, which activates PERK- and ATF6-dependent pathways that transiently limit CHOP-mediated apoptosis. Collectively, these findings underscore that the balance between adaptive and maladaptive UPR signaling critically determines renal outcome following injury [71].

In CKD, persistent ER stress becomes pathogenic. Sustained CHOP activation promotes inflammatory signaling and cytokine production, including IL-1β and IL-18, thereby driving fibrotic remodeling, nephron loss, and progressive deterioration of renal function. Through its extensive and dynamic organization, the ER establishes close contacts with other intracellular organelles—including mitochondria, endosomes, lysosomes, peroxisomes, the Golgi apparatus, and the plasma membrane—through MCSs, regions where membranes are closely apposed without fusion [1]. These dynamic structures allow the direct exchange of ions and lipids and contribute to the coordinated regulation of organelle function [75].

### 3.2. ER–Mitochondria Crosstalk

**Figure 2:**
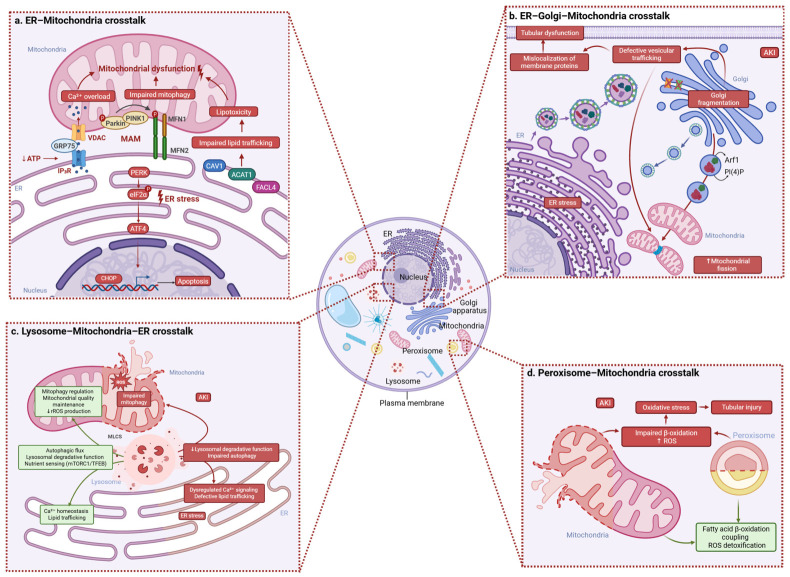
**Interplay of intracellular organelles in renal physiology and disease**. (**a**) **ER–Mitochondria crosstalk**. ER–mitochondria communication is mediated by mitochondria-associated membranes (MAMs), dynamic contact sites that regulate Ca^2+^ transfer, lipid trafficking, mitochondrial quality control, and stress responses. In renal injury, disruption of MAM integrity promotes mitochondrial dysfunction and ER stress. Dysregulated Ca^2+^ transfer through the IP3R–GRP75–VDAC axis induces mitochondrial Ca^2+^ overload and apoptosis, while altered MFN2 signaling contributes to defective mitochondrial homeostasis. In parallel, impaired lipid trafficking involving CAV1, ACAT1, and FACL4 promotes lipotoxicity and mitochondrial dysfunction. MAMs also regulate mitophagy through the PINK1–parkin pathway, and disruption of these quality control mechanisms contributes to accumulation of damaged mitochondria and progression of tubular injury in AKI and CKD. (**b**) **ER–Golgi–Mitochondria crosstalk**. The Golgi apparatus plays a central role in protein maturation and vesicular trafficking while communicating with the ER and mitochondria. During AKI, Golgi fragmentation and trafficking defects disrupt protein processing and membrane protein localization, leading to loss of epithelial polarity and tubular dysfunction. In this context, altered vesicular trafficking and Golgi-derived Arf1/PI(4)P signaling may exacerbate pathological mitochondrial fission and contribute to cellular stress responses. (**c**) **Lysosome–Mitochondria–ER crosstalk**. Lysosomes act as central signaling hubs coordinating interactions with mitochondria and the ER through membrane contact sites, regulating mitophagy, mitochondrial quality control, Ca^2+^ homeostasis, and lipid trafficking to sustain autophagic flux; during AKI, lysosomal dysfunction disrupts this crosstalk, impairing mitophagy, promoting ROS accumulation and ER stress, and ultimately contributing to tubular injury. (**d**) **Peroxisome–Mitochondria crosstalk**. Peroxisomes and mitochondria coordinate fatty acid β-oxidation and redox homeostasis through metabolic crosstalk, supporting cellular redox balance; disruption of this interplay during AKI impairs β-oxidation, increases ROS production, and promotes oxidative stress and tubular injury. Blue and black lines: physiological conditions; red lines: AKI, AKI-to-CKD conditions. Abbreviations: AKI: acute kidney injury; CKD: chronic kidney disease; MAMs: mitochondria-associated membranes; IP3R: inositol 1,4,5-trisphosphate receptor; GRP75: glucose-regulated protein 75; VDAC: voltage-dependent anion channel; MFN1: mitofusin 1; MFN2: mitofusin 2; PERK: protein kinase RNA-like endoplasmic reticulum kinase; eIF2α: eukaryotic initiation factor 2 alpha; ATF4: activating transcription factor 4; CHOP: C/EBP homologous protein; PINK1: PTEN-induced kinase 1; CAV1: caveolin-1; ACAT1: Acyl-CoA:cholesterol acyltransferase 1; FACL4: fatty acid CoA ligase 4; ARF1: ADP-ribosylation factor 1; PI(4)P: phosphatidylinositol 4-phosphate; MLCS: mitochondria–lysosome contact sites. Figures were created with BioRender.com.

Among MCSs, ER–mitochondria contacts are particularly relevant. As already described, MAMs are specialized ER domains that form close contacts with the OMM. These dynamic interfaces, composed of ER subdomains, OMM, and regulatory proteins, are involved in multiple processes, including Ca^2+^ transfer, lipid metabolism, ER stress responses, mitochondrial quality control, autophagy, and inflammasome assembly [76,77]. Their organization depends on cell type and pathophysiological conditions, and alterations have been described in different kidney diseases, including diabetic kidney disease (DKD) and AKI [78,79,80]. Under physiological conditions, MAMs enable Ca^2+^ transfer from the ER to mitochondria, thereby supporting cellular energy metabolism and mitochondrial function. This flux is primarily mediated by inositol 1,4,5-trisphosphate receptors (IP3Rs) located on the ER, which are functionally coupled to voltage-dependent anion channels (VDAC) on the OMM through the chaperone protein GRP75 [81,82]. IP3R activity is regulated by IP_3_, Ca^2+^, and ATP, which contributes to maintaining a balance between channel activation and the prevention of excessive Ca^2+^ release, stabilizing closed states [83]. In the kidney, this system is dysregulated during IRI, where ATP depletion and subsequent IP3R activation lead to increased intracellular Ca^2+^ levels, mitochondrial Ca^2+^ overload, and tubular cell apoptosis [84]. In addition to the IP3R–GRP75–VDAC1 axis, other MAM-associated proteins contribute to Ca^2+^ regulation, including mitofusins. In particular, mitofusin 2 (MFN2) is localized on both the ER and the OMM, whereas mitofusin 1 (MFN1) is found on the OMM. MFN2 plays a key role in maintaining ER–mitochondria contacts through the formation of homo- and heterodimers with MFN1 and participates not only in Ca^2+^ homeostasis but also in mitochondrial fusion, lipid metabolism, and ER stress responses [85,86] (Figure 2a). In an interesting, albeit preliminary, study conducted in a rat model of IRI, MFN2 overexpression was associated with reduced mitochondrial damage, decreased Ca^2+^ accumulation, and significantly lower levels of renal injury biomarkers, including BUN, creatinine, and NGAL. Despite its experimental limitations, the study suggests a renoprotective role for MFN2 in mitigating apoptosis and highlights the importance of proper endoplasmic reticulum–mitochondria coupling for the preservation of mitochondrial function [87]. Beyond Ca^2+^ signaling, MAMs also serve as key platforms for non-vesicular lipid trafficking between the ER and mitochondria. This process is regulated by lipid transfer proteins, including VAPB-PTPIP51, ORP5/8, MFN2, and caveolin-1 (CAV1), as well as enzymes such as acyl-CoA:cholesterol acyltransferase 1 (ACAT1) and fatty acid CoA ligase 4 (FACL4), which are involved in cholesterol and fatty acid metabolism [86,88,89,90,91,92]. Disruption of these mechanisms has been linked to various kidney diseases. In particular, CAV1 dysregulation impairs lipid trafficking and mitochondrial function and has been associated with DKD and IRI [93,94]. Increased ACAT1 expression is associated with lipid accumulation and renal damage, whereas its inhibition reduces lipotoxicity in DKD models [95]. Similarly, FACL4 contributes to tubular injury through ferroptosis, while its inhibition exerts protective effects in models of AKI and CKD [96,97,98]. MAMs also play a critical role in maintaining mitochondrial quality by regulating mitochondrial dynamics and mitophagy, which is primarily controlled by the PINK1–parkin signaling pathway. Under conditions of mitochondrial damage, PINK1 accumulates on the mitochondrial membrane and phosphorylates MFN2, promoting parkin recruitment and ubiquitination of mitochondrial proteins, thereby initiating the removal of damaged mitochondria [99]. However, this process may also lead to dissociation of MFN2 complexes and loss of ER–mitochondria contacts [100]. Another relevant study highlights the importance of mitochondrial bioenergetic efficiency as a key regulator of cellular homeostasis. Fatty acid-binding protein 4 (FABP4) has been identified as a mediator of ischemic and toxic AKI through the modulation of ER stress. In murine models, FABP4 deficiency significantly attenuated renal dysfunction, reducing serum creatinine and blood urea nitrogen levels while improving tubular injury, suggesting a pathogenic role for FABP4 in AKI progression. In this context, both MFN2 and FABP4 may represent promising therapeutic targets in AKI-related pathologies [101].

Mitophagy, a selective form of autophagy responsible for the removal of damaged or dysfunctional mitochondria, plays a protective role against oxidative stress, mitochondrial dysfunction, and inflammation. It also contributes to erythropoietin (EPO) regulation by attenuating inflammatory signaling, including inflammasome activation. Mitophagy is mediated through the PTEN-induced kinase 1/parkin (PINK1–parkin) pathway or receptor-dependent mechanisms involving proteins such as BCL2 interacting protein 3-like (BNIP3L/NIX), FUN14 domain-containing 1 (FUNDC1), prohibitin 2, and FKBP prolyl isomerase 8 (FKBP8). Among these, FUNDC1 is highly expressed in the kidney and is particularly relevant under hypoxic conditions, where it contributes to EPO production during stress erythropoiesis. Since the kidney is the primary site of EPO synthesis, impaired FUNDC1-mediated mitophagy may have important pathological consequences. Experimental studies in murine models have shown that FUNDC1 deficiency, including cisplatin-induced impairment, disrupts the clearance of damaged mitochondria, enhances inflammation, reduces EPO production, and exacerbates renal anemia, a common complication of CKD. Interestingly, while FUNDC1 appears dispensable for programmed mitochondrial elimination during terminal erythroid maturation, both FUNDC1 and NIX are required for mitochondrial remodeling during cardiac lineage differentiation. Although the role of mitophagy in renal erythropoietin-producing cells (REPs) remains incompletely understood, current evidence supports its essential function in maintaining mitochondrial homeostasis, including mitochondrial fission and fusion, quality control, organelle turnover, and inter-organelle communication. Mechanistically, enhanced inflammatory responses in REPs may compromise mitophagy, leading to insufficient EPO production. Collectively, these findings suggest that defective mitophagy contributes to impaired EPO synthesis in CKD and underline the importance of mitochondrial quality control in protecting REPs under stress conditions [102,103]. Targeting mitochondrial quality control pathways within the cardio–renal axis, an area still poorly explored, may therefore represent a promising therapeutic strategy. Mitochondrial proteases involved in proteostasis and organelle integrity are of particular interest. Among them, high-temperature requirement protein A2 (HtrA2/Omi) has emerged as an important regulator of mitochondrial quality control through its involvement in mitochondrial dynamics and morphology maintenance. Furthermore, HtrA2 contributes to inflammasome regulation by promoting late-stage autophagy and facilitating ASC oligomer turnover under stress conditions. Conversely, loss of HtrA2 activity within the mitochondrial intermembrane space has been associated with neurodegeneration and aging [104,105].

In DKD, hyperglycemia impairs these mechanisms by reducing PINK1–parkin and MFN2 expression, thereby disrupting mitophagy in proximal tubular cells [106].

### 3.3. Therapeutic Potential Approach

Experimental mouse model of sepsis-induced AKI, demonstrate that suppression of PERK/CHOP signaling alleviates kidney injury [74]. Chemical chaperones such as BiP inducer X (BIX) enhance ER folding capacity and reduce histological damage following IRI. Interestingly, depletion of tissue inhibitors of metalloproteinase 2 (TIMP2), an AKI biomarker and cell cycle arrest regulator, reduced ER stress and alleviated sepsis-induced kidney injury.

## 4. The Golgi Apparatus

### 4.1. Golgi Apparatus Transcriptional and Metabolic Regulation in Kidney

The Golgi apparatus is a membrane-bound organelle responsible for the post-translational modification, maturation, and sorting of proteins and lipids received from the endoplasmic reticulum (ER). It is composed of a series of flattened membrane cisternae arranged into cis, medial, and trans compartments, reflecting the directional processing of cargo along the secretory pathway. Structurally, the Golgi exhibits pronounced functional polarity, with a cis-face oriented toward the ER, a medial region, and a trans-face directed toward the plasma membrane. As proteins traverse the Golgi stack, they undergo multiple post-translational modifications, including glycosylation, sulfation, phosphorylation, and proteolytic processing, before being sorted to lysosomes, secretory vesicles, or specific plasma membrane domains. Bidirectional trafficking between the ER and Golgi is mediated by COPII-coated vesicles, which drive anterograde transport from the ER to the Golgi, and COPI-coated vesicles, which primarily support retrograde transport within Golgi compartments and back to the ER. The coordinated action of COPI and COPII vesicle coats ensures efficient cargo transport and maintenance of Golgi structure and function.

In renal cells, particularly tubular epithelial cells, the Golgi apparatus plays a critical role in the maturation and trafficking of membrane proteins, including transporters, ion channels (e.g., Ca^2+^ channels), and receptors. Proper Golgi function ensures accurate post-translational modification and polarized delivery of membrane proteins to either the apical or basolateral membrane, thereby maintaining epithelial polarity. This process is tightly integrated with ER function and vesicular trafficking pathways, particularly through the trans-Golgi network (TGN), which serves as a major sorting hub for outgoing cargo. Trafficking within this system is regulated by small GTPases, SNARE proteins, and cytoskeletal components [107].

Recent evidence further indicates that the Golgi apparatus participates in specific cellular stress responses. In mammals, Golgi stress signaling has been associated with several pathways, including the TFE3, CREB3, HSP47, proteoglycan and mucin (PG/Mucin), PERK, and MAPK pathways, which collectively influence cell fate decisions such as survival, apoptosis, and adaptive changes in glycosylation. In addition, the structural integrity of the Golgi ribbon—maintained by proteins such as GRASP65 and GRASP55—is dynamically regulated under stress conditions. Activation of kinases including Cdc2, GSK3β, RAF/MEK1/ERK1c, Plk1, and Plk3 promotes reversible Golgi fragmentation, a process that is increasingly recognized as an important component of the cellular stress response [107].

### 4.2. Role of Golgi Apparatus in Renal Pathogenesis

Golgi fragmentation is recognized as an early event in injured tubular epithelial cells and can significantly disrupt the fidelity of protein processing and trafficking. This structural disorganization promotes the mislocalization of ion transporters and other membrane proteins, thereby contributing to the loss of epithelial polarity and impairment of tubular function. In addition, defects in vesicular trafficking further exacerbate tubular dysfunction and may facilitate the progression of renal injury.

In CKD, persistent alterations of Golgi organization are associated with long-term cellular remodeling and the development of fibrosis. Increasing evidence suggests that, in this context, the Golgi apparatus is not merely a passive target of cellular damage but actively contributes to pathogenic processes that sustain inflammation, fibrogenic signaling, and apoptotic responses. Consequently, Golgi dysfunction is emerging as a relevant component of the cellular mechanisms underlying the progression of chronic renal disease.

### 4.3. Crosstalk with Golgi Apparatus

Experimental studies suggest that protecting cytoskeletal integrity and regulating proteins involved in membrane dynamics may reduce Golgi fragmentation. Another potential strategy is controlling the secretion of pro-inflammatory or pro-fibrotic mediators through modulation of intracellular trafficking pathways.

Rather than functioning as isolated compartments, the ER, Golgi apparatus, and mitochondria form an integrated stress response network that determines renal cell fate. Coming from ER–mitochondria contact sites, sites known as mitochondria-associated membranes (MAMs) regulate calcium transfer and bioenergetics. PERK participates in ER mitochondria crosstalk and may promote stress-induced mitochondrial hyperfusion (SIMH), a transient pro-survival mechanism enhancing ATP production during acute injury. Moreover, the IRE1α–XBP1 axis modulates both adaptive proteostasis and inflammatory signaling. Prolonged activation may engage ASK1–JNK pathways, amplifying cytokine production and apoptosis. In diabetic kidney disease (DKD), disruption of MAM integrity and downregulation of Mfn2 enhance PERK signaling and podocyte apoptosis [108]. Restoration of Mfn2 attenuates mitochondrial dysfunction and ER stress, highlighting the therapeutic relevance of the Mfn2–PERK axis [78]. Similarly, ER–Golgi communication is essential for proper ATF6 activation, while Golgi dysfunction can exacerbate ER stress, creating a feed-forward loop that amplifies cellular injury.

The interconnectivity between the ER, the Golgi apparatus, and mitochondria has the role to sustain a pro-survival cellular response to external stressors such as excess of substrates (e.g., glucose, lipids) or misfolded protein concentration or in fatty acids, oxidative stress, iron imbalance, viral infections, and hypoxia (known as important causes of ER stress) [56]. Recent studies demonstrated the implication of ER stress and UPR system in the pathophysiology of AKI, CKD, DKD, glomerulonephritis, and glomerulopathies associated with genetic mutations.

In DKD, an altered functional crosstalk between ER and mitochondria, mediated by MAMs, significantly contributes to podocyte injury and disease progression. Under high-glucose (HG) conditions, mitochondrial dysfunction, a pivotal event in DKD, is associated with MAMs disruption, increased apoptosis, PERK pathway activation, and downregulation of Mfn2. Given that Mfn2 physically interacts with PERK, HG exposure reduces the Mfn2–PERK interaction, thereby promoting ER stress signaling. Consistently, Mfn2 silencing in podocytes induces mitochondrial dysfunction, MAMs reduction, PERK pathway activation, and enhanced apoptosis. Conversely, Mfn2 overexpression markedly attenuates all HG-induced alterations. Thus, the Mfn2–PERK signaling axis, as a critical regulator of ER–mitochondria crosstalk in DKD, may represent a promising therapeutic target for preventing podocyte injury.

### 4.4. Therapeutical Potential Approach

ER stress and organelle crosstalk represent dynamic regulatory systems rather than linear apoptotic cascades. Given its vital role in renal injury, modulation of ER stress and UPR pathways represents a promising therapeutic strategy. The degree, duration, and cellular context of UPR activation determine whether renal cells adapt or undergo apoptosis. Future research should focus on several aspects like characterizing ER stress biomarkers in AKI patients, developing strategies that selectively inhibit pro-apoptotic mediators such as CHOP (involved in different pathways activated by UPR), and exploring the combined targeting of ER, mitochondria, and Golgi signaling networks.

A system-level understanding of communication between organelles could enable precision medicine approaches capable of shifting the balance from maladaptive stress signaling toward kidney recovery and determining the exact moment to administer drugs or molecules that activate or inhibit signaling to prevent nephron failure.

## 5. Mitochondria

### 5.1. Mitochondrial Transcriptional and Metabolic Regulation in Kidney

Mitochondria are double-membrane-bound organelles that play a central role in cellular homeostasis. In addition to their classical function as the primary site of ATP production through oxidative phosphorylation, mitochondria act as key hubs of metabolic integration, coordinating fundamental pathways such as the tricarboxylic acid (TCA) cycle, fatty acid β-oxidation, and amino acid metabolism [109]. Beyond metabolism, mitochondria regulate essential signaling processes, including reactive oxygen species (ROS) generation, Ca^2+^ homeostasis, and the intrinsic apoptotic pathway [110].

Mitochondrial integrity is preserved through tightly regulated quality control mechanisms, including fusion and fission dynamics and mitophagy. Controlled fusion promotes content mixing and functional complementation, whereas fission enables proper organelle distribution and the segregation of damaged mitochondria, which are subsequently removed by mitophagy, a selective form of autophagy [111]. These processes collectively ensure efficient bioenergetics, stress adaptation, and cell survival.

### 5.2. Role of Mitochondria in Renal Pathogenesis

The kidney is one of the most energy-demanding organs, and renal cells, particularly PTECs and podocytes, are highly enriched in mitochondria to sustain their metabolic requirements [112]. In this context, mitochondria play a pivotal role in regulating Ca^2+^ signaling, ROS production, and metabolic flexibility. While physiological ROS generation participates in intracellular signaling, excessive ROS accumulation induces oxidative damage to lipids, proteins, and mitochondrial DNA (mtDNA), ultimately impairing cellular function [113,114]. Similarly, mitochondrial Ca^2+^ overload can trigger permeability transition, apoptotic signaling, and cell death [115].

Impaired mitophagy exacerbates mitochondrial dysfunction by allowing the accumulation of damaged organelles, thereby amplifying cellular stress and inflammatory responses [116]. Moreover, disruption of the balance between mitochondrial fusion and fission is a hallmark of kidney injury [112,117]. Reduced expression or dysfunction of MFN2 is frequently observed in metabolic and diabetic disorders, leading to mitochondrial fragmentation, defective organelle turnover, and increased susceptibility to apoptosis [118]. Conversely, hyperactivation of dynamin-related protein 1 (DRP1) promotes excessive fission and has been linked to tubular injury, inflammation, and progressive renal dysfunction [119]. Together, these alterations identify mitochondrial dynamics as a central pathogenic node in kidney disease [120]. During IRI-induced AKI, multiple pathways converge to promote mitophagy. Hypoxia-inducible factor 1α (HIF-1α) upregulates BNIP3, which anchors to the OMM and interacts with LC3 on autophagosomes, facilitating mitochondrial clearance [121]. Under ischemic conditions, loss of mitochondrial membrane potential leads to PINK1 accumulation on the outer membrane, where it recruits and activates parkin, further promoting mitophagy. Moreover, phosphorylation of Drp1 at Ser616 enhances its activation and translocation to mitochondria, contributing to mitochondrial fragmentation and subsequent mitophagy.

### 5.3. Crosstalk with Mitochondria

Rather than functioning in isolation, mitochondria act as central integrators of metabolic, redox, and cell death signals through extensive inter-organelle communication. Tight coordination with other cellular compartments is essential for renal homeostasis and decisively shapes cellular fate in health and disease [122,123]. Mitochondrial dysfunction in the kidney often arises from profound alterations in this inter-organelle network, particularly involving the ER, lysosomes, peroxisomes, the Golgi apparatus, and the nucleus [124].

The best-characterized interaction is the crosstalk with the ER via MAMs, which regulate Ca^2+^ transfer, lipid metabolism, and stress signaling [125]. Pathological modulation of MAMs directly contributes to kidney disease progression. Aberrant enhancement of ER–mitochondria contacts has been shown to promote ER stress, mitochondrial dysfunction, and tubulointerstitial fibrosis in diabetic kidney disease [126], while disruption of the MAPK1–PACS-2 axis impairs MAM integrity and mitochondrial bioenergetics in tubular epithelial cells [127]. ER–mitochondria crosstalk is also tightly linked to regulated cell death, including ferroptosis, through pathways such as PERK/ATF4/CHAC1 [128]. Conversely, restoration of MAM integrity—via MFN2 upregulation or VDR activation—enhances mitophagy and attenuates renal injury in both chronic and acute settings [87,129].

Mitochondria additionally communicate with lysosomes through mitophagy-dependent quality control mechanisms [130] and with peroxisomes to coordinate β-oxidation and redox homeostasis [131]. Emerging evidence also implicates the Golgi apparatus in mitochondrial dynamics, as Golgi-derived vesicles and PI(4)P-dependent signaling participate in mitochondrial fission [132,133]. Finally, damaged mitochondria signal to the nucleus through mtDNA and ROS release, activating inflammatory and antioxidant transcriptional programs, including cGAS–STING and Nrf2 pathways, whose dysregulation contributes to CKD progression [36,40,134,135,136,137].

### 5.4. Therapeutical Potential Approach

The growing body of evidence supports a paradigm shift in renal pathophysiology in which mitochondrial dysfunction is no longer viewed as an isolated defect but as the outcome of disrupted inter-organelle communication. Consequently, therapeutic strategies aimed at restoring mitochondrial homeostasis increasingly focus on improving mitochondrial quality control, bioenergetics, and crosstalk with other organelles. Potential approaches include modulation of mitochondrial dynamics (e.g., MFN2 stabilization or DRP1 inhibition), enhancement of mitophagy, restoration of MAM integrity, and activation of mitochondrial biogenesis through the PGC-1α axis. Impaired mitochondrial biogenesis, driven by reduced PGC-1α activity, further exacerbates metabolic dysfunction in kidney disease [22,137,138]. In this context, mitochondrial transplantation (MITO) has emerged as a promising strategy to restore bioenergetics and cellular homeostasis in the transplantation setting, particularly for marginal grafts [139]. Preclinical evidence shows that MITO improves ATP production, reduces oxidative stress and inflammation, and preserves mitochondrial structure in ischemic kidney injury [140]. By targeting mitochondria-centered signaling networks, these strategies may not only preserve mitochondrial function but also improve ER stress responses, lysosomal clearance, and nuclear transcriptional adaptation, ultimately mitigating tubular injury and limiting progression from AKI to CKD.

## 6. Lysosomes

### 6.1. Lysosomal Transcriptional and Metabolic Regulation in Kidney

Lysosomes are intracellular organelles bounded by a single membrane and are primarily responsible for the degradation and recycling of cellular macromolecules through the action of a broad repertoire of hydrolytic enzymes. These enzymes function optimally in an acidic environment (pH 4.5–5.5), which is maintained by the lysosomal multi-subunit V-ATPase that transports H^+^ ions into the lysosomal lumen and allows the activation of more than 50 hydrolases [141]. Through this degradative capacity, lysosomes are essential for preserving cellular homeostasis by supporting autophagy, endocytosis, and the clearance of damaged or unnecessary cellular components [142].

Beyond their catabolic role, lysosomes act as dynamic hubs integrating metabolic and stress signals. They contribute to cellular adaptation by coordinating nutrient sensing, energy homeostasis, and stress responses and by interacting with other intracellular organelles such as mitochondria and the endoplasmic reticulum (ER), thereby supporting mutual homeostatic regulation [143]. In highly specialized organs such as the kidney, where epithelial cells must cope with constant metabolic and environmental stress, lysosomal integrity is particularly critical for maintaining cellular function.

### 6.2. Role of Lysosomes in Renal Pathogenesis

Maintenance of cellular homeostasis is essential for normal kidney function, which relies on distinct cell populations to regulate nutrient absorption, waste elimination, acid–base balance, osmoregulation, blood pressure, and hormone secretion. In renal tubular epithelial cells (TECs), lysosome-dependent autophagy plays a crucial protective role in response to stress conditions such as proteinuria, IRI, and AKI.

Experimental evidence from murine models of ischemia–reperfusion injury and septic AKI has demonstrated that autophagy promotes TEC survival during injury and preserves nephron morphology [144,145]. In particular, studies using tubular-specific autophagy-deficient mice (Atg5/Atg7 knockout models) have shown that impaired autophagy exacerbates tubular injury and delays renal recovery following ischemic stress [144].

In experimental models of sepsis-induced AKI, impairment of autophagic flux contributes to proximal tubular damage, whereas preservation of autophagy limits cell death and renal dysfunction [145]. However, most mechanistic insights currently derive from animal models or immortalized tubular epithelial cell lines, while direct evidence in human AKI remains limited. Moreover, the extent to which experimental modulation of autophagy recapitulates the complexity and heterogeneity of human kidney injury is still incompletely understood.

Multiple studies have demonstrated that autophagy promotes TEC survival during injury and preserves nephron morphology [146]. In experimental models of sepsis-induced AKI, autophagy impairment contributes to proximal tubular damage, whereas preservation of autophagic flux prevents cell death and renal dysfunction [145]. Autophagy proceeds through sequential steps, including phagophore formation, autophagosome maturation, fusion with lysosomes, and autolysosomal degradation, a process tightly regulated by autophagy-related (Atg) proteins and nutrient availability.

Dysregulation of lysosomal function and autophagic flux is now recognized as a major contributor to AKI pathogenesis and maladaptive repair leading to CKD. In TECs, lysosomal depletion or dysfunction impairs autophagosome clearance, resulting in the accumulation of damaged organelles, inflammasome activation, and profibrotic signaling, ultimately delaying epithelial regeneration and exacerbating tubular injury [147]. In this context, impaired lysosomal signaling represents a key driver of renal disease progression.

### 6.3. Crosstalk with Lysosomes

Lysosomes play a pivotal role in coordinating intracellular stress responses through complex signaling pathways and inter-organelle communication. Autophagy initiation is regulated by energy-sensing kinases such as AMPK and mTORC1, which converge on the ULK1–Atg13–FIP200 complex [148]. During nutrient deprivation or stress, mTORC1 inhibition enables ULK1 activation and downstream recruitment of autophagic machinery, ultimately leading to lysosome-dependent cargo degradation.

Lysosomes also serve as central nodes for transcriptional regulation via transcription factor EB (TFEB), a master regulator of lysosomal biogenesis and autophagy. Under basal conditions, mTORC1 phosphorylates TFEB on the lysosomal surface, retaining it in the cytoplasm and suppressing transcriptional activity. Under stress, TFEB translocates to the nucleus to activate genes involved in lysosome formation and autophagosome–lysosome fusion [59]. In experimental models of AKI, persistent mTORC1 activity prevents TFEB nuclear translocation, impairing lysosomal function and autophagic clearance, thereby aggravating cellular injury [149]. Nevertheless, whether TFEB dysregulation acts as a primary pathogenic driver or represents a secondary adaptive response during human AKI remains to be fully clarified. Although TFEB activation is generally considered protective in acute injury settings, sustained or excessive activation of TFEB-mediated autophagy has also been associated with maladaptive repair and tubulointerstitial fibrosis in experimental CKD models, suggesting that the timing and magnitude of lysosomal activation critically influence disease outcomes [150].

Beyond signaling, lysosomes physically interact with mitochondria and the ER through specialized membrane contact sites. These lysosome-centered interactions regulate mitochondrial quality control, calcium homeostasis, and lipid trafficking. Disruption of lysosome–mitochondria or ER–lysosome communication exacerbates mitochondrial dysfunction, reactive oxygen species accumulation, and ER stress, all of which contribute to tubular cell injury in kidney disease [151,152,153,154]. Thus, lysosomal crosstalk integrates metabolic stress with organelle dysfunction and determines renal cell fate.

### 6.4. Therapeutical Potential Approach

Given the central role of lysosomes in autophagy, metabolic signaling, and inter-organelle communication, targeting lysosome-centered pathways represents a promising therapeutic strategy in kidney disease. Pharmacological modulation of lysosomal signaling aims to restore autophagic flux, enhance organelle clearance, and improve cellular stress resilience.

Strategies include inhibition of kinases that retain TFEB in the cytoplasm, such as mTORC1, or direct activation of TFEB nuclear translocation using small-molecule compounds, including genistein [155]. By restoring lysosomal biogenesis and function, these approaches may indirectly improve mitochondrial quality control, attenuate ER stress responses, and limit tubular injury. Consequently, therapeutic modulation of lysosome-centered signaling holds strong potential to mitigate AKI severity and slow the progression toward CKD [156].

## 7. Peroxisomes

### 7.1. Transcriptional and Metabolic Peroxisome’s Program in Kidney

Peroxisomes are single-membrane-bound organelles ranging from 0.1 to 0.5 µm in size, present in most eukaryotic cells, with the highest abundance in the liver and kidneys [157]. They have specific proteins known as peroxins, encoded by PEX genes. Peroxisomal membrane proteins are recognized and bound by the cytosolic protein PEX19 through a mitochondrial-peroxisomal targeting signal (mPTS). PEX19 interacts with PEX3, which is anchored to the peroxisomal membrane, helping the release and incorporation of transported proteins into the membrane, before being recycled back into the cytoplasm. Peroxisomes play a vital role in human health and have biochemical properties that make them valuable in various biotechnological applications. A typical human cell has between 100 and 1000 peroxisomes distributed throughout the cytoplasm, forming contacts with other organelles, particularly the ER, lipid droplets, and mitochondria [157,158]. The extensive metabolic functions of peroxisomes require efficient transport systems for substrates and cofactors, especially for two primary functions: FAO and hydrogen peroxide metabolism. The renal cortex, particularly proximal tubules, heavily relies on β-oxidation as the primary energy source for transport systems in this nephron segment [157]. Peroxisomes closely cooperate with mitochondria in energy metabolism: mitochondria preferentially oxidize short- and medium-chain fatty acids, while peroxisomes metabolize very long-chain fatty acids (VLCFAs), shortening them to allow mitochondrial oxidation into acetyl-CoA. Since peroxisomes lack respiratory chain enzymes, peroxisomal β-oxidation does not directly contribute to ATP production but instead releases most energy as heat. Peroxisomal β-oxidation is unique in that it generates H_2_O_2_ as a byproduct of oxidative reactions. Studies have shown that impaired β-oxidation worsens renal injury, while efficient function prevents fatty acid accumulation, lipid peroxidation, and the formation of lipid aldehydes, which can further aggravate renal damage. To date, approximately 50 peroxisomal enzymes have been shown to take part in essential metabolic processes such as fatty acid β-oxidation, either phospholipid biosynthesis, and reactive oxygen species (ROS) metabolism. These functions make peroxisomes indispensable for human health and development. Peroxisomes are highly dynamic organelles capable of rapid assembly, proliferation, and degradation in response to metabolic demands. In response to oxidative stress, the ATM signaling pathway activates ULK1 kinase and inhibits mTORC1 to induce autophagy [158]. Peroxisome-specific autophagy (pexophagy) is regulated by ATM-mediated phosphorylation of PEX5 at Ser141, promoting mono-ubiquitination at Lys209. This modification allows ubiquitinated PEX5 to be recognized by the adaptor protein P62, directing the autophagosome to peroxisomes for degradation [157,158].

### 7.2. Role of Peroxisomes in Renal Pathogenesis

Peroxisomes are particularly abundant in proximal tubules, especially in segments S2 and S3, while the S1 segment lacks these organelles [157,159]. Their presence is negligible in glomeruli, distal tubules, and collecting ducts. During tubular regeneration following AKI, mitochondria, and the ER appear before peroxisomes, which regenerate later alongside lysosomes. Proximal tubular cells are primary targets of ischemic injury in AKI. Increased parenchymal levels of free fatty acids and reduced ATP levels are consistently seen in ischemic kidneys. ROS plays a crucial role in the pathophysiology of IRI. Reperfusion of the ischemic kidney leads to excessive ROS generation and worsening ischemic damage. Peroxisomal antioxidant mechanisms, including superoxide dismutase, glutathione peroxidase, and the key enzyme catalase, take part in ROS detoxification. Renal ischemia reduces FAO and catalase activity. The reperfusion phase following ischemia induces significant oxidative stress [157]. The accumulation of unmetabolized fatty acids, combined with inactivated and/or degraded catalase, promotes lipid peroxidation and cellular damage. There are two mechanisms for peroxisome biogenesis: budding from the ER and multiplication through elongation and fission. The Pex11 gene family is involved in peroxisome elongation, a critical initial step in their division [159]. Among the three Pex11 subtypes (a, b, and c), Pex11a and Pex11c are primarily expressed in the kidney and liver, while Pex11b is ubiquitous.

Pex11a deficiency impairs peroxisome elongation, reduces peroxisomal abundance, and disrupts peroxisomal fatty acid oxidation, leading to lipid accumulation in the liver (steatosis) or kidneys (CKD) [159]. Increased butyrate availability (achieved through direct supplementation with butyrate-producing probiotics and dietary fiber) induces Pex11a expression and enhances peroxisomal fatty acid oxidation genes, increasing peroxisome abundance and improving lipid metabolism [160].

### 7.3. Crosstalk with Peroxisomes

Peroxisomes maintain essential crosstalk with the ER and mitochondria via MCS to preserve redox balance during acute renal stress. In AKI, particularly during IRI, the decoupling of peroxisomal–mitochondrial fatty acid β-oxidation leads to an acute accumulation of toxic lipids and a surge in ROS [157,161]. These organelles collaborate in a “multi-organelle defense layer,” where peroxisomes neutralize mitochondrial-derived ROS to prevent acute tubular necrosis [158,162]. Furthermore, ER–peroxisome contacts are critical for the rapid organelle biogenesis needed during the tubular repair phase post-insult [158,161]. Sustaining this inter-organelle constructive interaction is vital to mitigate acute injury and help renal recovery [162].

### 7.4. Therapeutical Potential Approach

Systemic dysfunction induced by impaired peroxisomal biogenesis, such as Pex11 deficiency in proximal tubular cells, highlights the therapeutic relevance of peroxisome–organelle crosstalk in CKD progression. In Pex11a knockout mice, the reduction in functional peroxisomes leads to severe metabolic dysregulation, which becomes particularly evident under deoxycorticosterone acetate (DOCA)-salt treatment, a model of hypertension-driven renal injury [159]. Compared with wild-type animals, Pex11a−/− mice display markedly aggravated tubulointerstitial damage, including enhanced tubular lipid accumulation, increased albuminuria and urinary N-acetyl-β-D-glucosaminidase excretion, elevated oxidative stress (urinary 8-iso-prostane), and more pronounced fibrosis and inflammatory infiltration. Mechanistically, these findings support a key role for peroxisomes in coordinating lipid metabolism, redox balance, and mitochondrial function in proximal tubular cells, thereby limiting lipotoxicity and injury propagation along the mitochondria–peroxisome axis.

Importantly, these data suggest that enhancing peroxisomal biogenesis or activity may represent a promising therapeutic strategy to attenuate proteinuria-driven tubular damage and slow CKD progression. However, the translational potential of this approach must be interpreted with caution [159,163,164]. The DOCA-salt model induces severe systemic hypertension and vascular inflammation, making it difficult to fully disentangle whether the observed renal phenotype in Pex11a-deficient mice is driven primarily by intrinsic tubular metabolic failure or by heightened susceptibility to systemic hemodynamic stress. Moreover, significant species-specific differences in peroxisomal biology exist between rodents and humans, including divergent metabolic rates, enzyme composition, and peroxisome proliferator responses, which may limit direct clinical extrapolation. Finally, CKD in humans is a slow, multifactorial process shaped by aging, diabetes, and vascular comorbidities, which is only partially captured by acute or accelerated experimental models. Therefore, while targeting peroxisome–mitochondria–ER crosstalk represents an attractive therapeutic avenue, future validation in human-relevant platforms such as kidney organoids and microfluidic kidney-on-chip systems will be essential to confirm its clinical feasibility.

## 8. Conclusions

Inter-organelle communication among the endoplasmic reticulum, mitochondria, nucleus, lysosomes, and peroxisomes is a central determinant of renal cell physiology. In AKI, this coordinated network is rapidly disrupted, leading to maladaptive nuclear reprogramming, ER stress, mitochondrial dysfunction, impaired autophagy, and peroxisomal redox imbalance. Persistent failure of organelle crosstalk prevents recovery and promotes fibro-inflammatory remodeling, driving the transition from AKI to CKD.

Defining how these interconnected organelle systems are renewed during injury and repair will be essential to understand disease progression. Targeting the preservation of organelle network integrity represents a promising strategy to enhance renal recovery and counteract CKD development.

## Data Availability

No new data were created or analyzed in this study. Data sharing is not applicable to this article.

## Abbreviations

The following abbreviations are used in this manuscript:

ACOX1	Acyl-CoA oxidase 1
AKI	Acute kidney injury
ARE	Antioxidant response element
ATM	Ataxia telangiectasia mutated
ATR	ATM- and Rad-3-related
Atg	Autophagy-related
ATF6	Activating transcription factor 6
BiP	Binding immunoglobulin protein
CKD	Chronic kidney disease
Cxcl12	C-X-C motif chemokine ligand 12
DDR	DNA damage response
DKD	Diabetic kidney disease
DOCA	Deoxycorticosterone acetate
DAMP	Damage-associated molecular pattern
eIF2α	Eukaryotic translation initiation factor 2 subunit alpha
ER	Endoplasmic reticulum
ERAD	ER-associated degradation
FAO	Fatty acid oxidation
GMP-AMP	Cyclic guanosine monophosphate-adenosine monophosphate
HG	High-glucose
HO-1	Heme oxygenase-1
IFN-I	Type I interferons
IKK	IκB kinase
IRE1α	Inositol-requiring enzyme 1α
IRI	Ischemia/Reperfusion injury
IRF3	Interferon regulatory factor 3
MAMs	Mitochondria-associated membranes
mPTS	Mitochondrial-peroxisomal targeting signal
mtDNA	Mitochondrial DNA
mTORC1	Mechanistic target of rapamycin complex 1
NQO1	Quinone oxidoreductase 1
PARP1	Poly(ADP-ribose) polymerase 1
PERK	Protein kinase R-like ER kinase
PGC-1α	Proliferator-activated receptor gamma coactivator 1-alpha
PTECs	Proximal tubular epithelial cells
RER	Rough ER
ROS	Reactive oxygen species
SER	Smooth ER
SIMH	Stress-induced mitochondrial hyperfusion
STING	Stimulator of interferon gene
TBK1	TANK-binding kinase 1
TCA	Tricarboxylic acid
TECs	Tubular epithelial cells
TFEB	Transcription factor EB
TGF-β	Transforming growth factor-β
TIMP2	Tissue inhibitors of metalloproteinase 2
UPR	Unfolded protein response
VLCFAs	Very long-chain fatty acids
